# Serum lactate dehydrogenase and rapidly progressive interstitial lung disease are associated with increased mortality in anti-melanoma differentiation-associated gene 5 antibody-positive dermatomyositis

**DOI:** 10.3389/fimmu.2025.1642682

**Published:** 2025-11-10

**Authors:** Gang Wang, Dong Yan, Yujie Zhang, Chang Liu, Zhichun Liu

**Affiliations:** Department of Rheumatology and Immunology, The Second Affiliated Hospital of Soochow University, Suzhou, Jiangsu, China

**Keywords:** lactate dehydrogenase, dermatomyositis, anti-MDA5 positive dermatomyositis, rapidly progressive interstitial lung disease, anti-MDA5 antibody, clinical characteristics, prognosis

## Abstract

**Objective:**

This study aims to evaluate the clinical characteristics and prognostic significance of serum lactate dehydrogenase (LDH) in patients with anti-MDA5+ dermatomyositis (anti-MDA5+ DM).

**Methods:**

We assessed 246 consecutive patients with anti-MDA5+ DM. The patients were divided into two groups based on LDH levels: the LDH ≤ 338 U/L group and the LDH > 338 U/L group. We compared the clinical characteristics, laboratory findings, and long-term prognosis between the two groups.

**Results:**

Overall, the one-year mortality rate in patients with anti-MDA5+ DM was high, at 24.39% (60/246). LDH levels exhibited a nonlinear, inverted S-shaped relationship with the overall mortality risk in anti-MDA5+ DM patients (nonlinear P = 0.001). Patients in the LDH > 338 U/L group had significantly higher levels of ALT [64.0 (32.0, 121.0) vs 40.0 (23.0, 66.5), P<0.001], AST [75.0 (47.5, 134.0) vs 40.0 (26.0, 60.6), P<0.001], CK [107.0 (42.0, 208.8) vs 50.0 (35.5, 100.5), P<0.001], CRP [8.5 (3.5, 17.6) vs 4.7 (2.7, 10.2), P<0.001], and serum ferritin levels [1307.6 (679.8, 1565.5) vs 1001.0 (391.2, 1307.6), P<0.001] compared to the LDH ≤ 338 U/L group. Additionally, the positivity rate of Anti-Ro52 antibodies (70.7% vs 57.7%, P = 0.033), the incidence of rapidly progressive interstitial lung disease (RPILD) (42.3% vs 29.3%, P = 0.033), and the mortality rate (35.0% vs 13.8%, P<0.001) were significantly higher in the LDH > 338 U/L group than in the LDH ≤ 338 U/L group. Multivariable regression analysis revealed that LDH > 338 U/L and the presence of RPILD were associated with poor prognosis [hazard ratios of 2.253 (95% CI 1.258, 4.035, P = 0.006) and 10.293 (95% CI 4.683, 22.623, P < 0.001), respectively].

**Conclusion:**

Patients with different LDH levels exhibit distinct clinical characteristics, laboratory findings, and long-term prognosis. Elevated LDH levels (> 338 U/L) and the presence of RPILD are associated with poor prognosis.

## Highlights

- Patients with anti-MDA5+ DM present different clinical features, laboratory test results, and long-term prognosis according to different levels of LDH.
- Elevated LDH levels (> 338 U/L) and the presence of RPILD are poor prognostic risk factors for patients with anti-MDA5+ DM.
- Understanding the clinical characteristics of these patients will help clinicians to develop individualised treatment plans for each patient.

## Introduction

Anti-melanoma differentiation-associated gene 5-positive dermatomyositis (anti-MDA5+ DM) is a special subtype of dermatomyositis (DM), characterized by a high incidence of rapidly progressive interstitial lung disease (RPILD) and poor prognosis, making clinical management extremely challenging (1, 2). Studies have shown that patients with anti-MDA5+ DM are prone to developing interstitial lung disease (ILD), with a probability ranging from 50% to 100% (3). A large cohort study from China reported that 54% of anti-MDA5+ DM patients have RPILD (4). Approximately 47% of anti-MDA5+ DM patients died from respiratory failure due to RPILD within one year of diagnosis, highlighting the urgency of prognosis evaluation and early intervention (5). In recent years, exploring reliable and easily accessible biomarkers to optimize risk stratification has become a research focus. For example, B cell activating factor (BAFF) and the neutrophil-to-lymphocyte ratio (NLR) have been confirmed to be associated with disease activity and mortality in anti-MDA5+ DM (6, 7). Plasma ferritin, as a marker of systemic inflammation, has also been incorporated into the prognosis evaluation system for anti-MDA5+ DM (8–11).

At present, there are still limitations in the research on biomarkers for anti-MDA5+ DM. First, some indicators [such as specific cytokines or gene expression profiles like Krebs von den Lungen-6 (KL-6) and the proportion of CD4+ CXCR4+ T cells] are complex to detect and costly, making them difficult to promote in primary care hospitals (12). Second, most existing studies focus on single biomarkers and lack systematic integration of multidimensional indicators (such as inflammation, organ damage, and immune features). Third, the conclusions of most studies are based on small sample sizes or single-center data, without incorporating multi-center data and insufficiently adjusting for confounding factors (such as treatment regimens and comorbidities), leading to limited generalizability of the results (7). In this context, lactate dehydrogenase (LDH) has become a potential breakthrough due to its unique clinical applicability. LDH, an enzyme widely involved in cellular metabolism, has elevated levels that can reflect tissue damage (such as muscles and lungs) or systemic inflammatory responses, and is closely associated with disease severity and prognosis in various autoimmune diseases (such as systemic lupus erythematosus and idiopathic inflammatory myopathies) (13–15). LDH testing is low in cost and widely available, but its prognostic value in anti-MDA5+ DM remains unclear, especially regarding the predictive efficacy of LDH and its dynamic changes on mortality risk, which still requires further investigation. Therefore, this study aims to evaluate the clinical characteristics and prognostic significance of serum LDH levels in patients with anti-MDA5+ DM, to help achieve accurate assessment of the patient’s condition and improve the prognosis of anti-MDA5+ DM patients.

## Methods

We conducted a retrospective cohort study of patients from the Nanjing Medical University Myositis-Associated Interstitial Lung Disease cohort. The Nanjing Medical University Myositis-Associated Interstitial Lung Disease cohort is a multicenter, retrospective, longitudinal regional cohort with data from ten tertiary hospitals in the East China region. This cohort has been described and used in several previous studies, and the amount of patient data is still growing and being used by clinicians (1, 16, 17). This study utilized the same patient cohort as our previously published research, but focused on a different research objective—the prognostic role of LDH (18).

A total of 246 consecutive patients with anti-MDA5+ DM were included in this study. The clinicians evaluated the patient’s clinical manifestations, laboratory tests, and recorded the patient’s follow-up information. The research variables included in the analysis of this study are as follows: (i) general demographic characteristics: gender, age, course of the disease (the time interval between onset of symptoms and diagnosis), follow-up period; (ii) clinical features: proximal muscle involvement (indicating proximal muscle weakness), skin rash, Gottron’s papules, mechanic’s hands, heliotrope rash, V sign, shawl sign, and skin ulcers; (iii) laboratory tests: aspartate aminotransferase (AST), alanine aminotransferase (ALT), lactate dehydrogenase (LDH), creatine kinase (CK), C-reactive protein (CRP), erythrocyte sedimentation rate (ESR), serum ferritin, anti-nuclear antibodies (ANA), anti-Ro52 antibodies, and anti-aminoacyl-tRNA synthetase (ARS) antibodies. All patients were tested for myositis-specific and related antibodies. Anti-Ro52 antibodies, anti-ARS antibodies, and anti-MDA5 antibodies were measured by immunoblotting, with anti-MDA5 titers categorized into three groups (MDA5+, MDA5++, MDA5+++, Euroimmun, Lubeck, Germany). ANA is detected using the indirect immunofluorescence assay (IIFAs) (17).

The diagnosis of DM was determined based on the 1975 Bohan/Peter criteria or Sontheimer’s criteria for polymyositis and DM classification criteria (19, 20). The presence of ILD and RPILD was evaluated via chest radiography or high-resolution computed tomography (HRCT). The diagnosis of ILD and RPILD is based on currently internationally accepted standards (21–23). The specific details of the diagnosis and assessment of ILD and RPILD in this cohort study have been described in detail in previous studies (1, 16, 17). The exclusion criteria are: (i) the presence of other autoimmune diseases such as systemic lupus erythematosus, rheumatoid arthritis, gout, ankylosing spondylitis, IgG4-related disease, systemic sclerosis, etc.; (ii) patients with missing or incomplete clinical data. All patients were followed up for 12 months, and if a patient died during the 1-year follow-up period, it was recorded as an adverse outcome endpoint. Overall survival is defined as the time from the initial diagnosis to the date of death or the last follow-up for anti-MDA5+ DM patients.

In this study, laboratory test results (e.g., lactate dehydrogenase) were obtained at the time of the patient’s initial diagnosis of anti-MDA5+ DM, which serves as the baseline data. The 12-month follow-up period corresponds to the first year from the initial consultation for all patients, and the prevalence of clinical manifestations (such as mechanic’s hands, RPILD, etc.) refers to the evaluation results at the time of the initial diagnosis.

The study protocol was approved by the Ethics Committee of the Second Affiliated Hospital of Soochow University (Ethics approval number: JD-HG-2023-09). This study was conducted in accordance with the local legislation and institutional requirements. Written informed consent for participation in this study was provided by the participants’ legal guardians/next of kin. All procedures were carried out in accordance with the declaration of Helsinki.

### Statistical analysis

The data in this study were analyzed using SPSS statistical software version 25.0 (IBM Corporation, New York, USA) and R software (version 4.2.1). For continuous variables, the Kolmogorov-Smirnov test and Levene’s test were first conducted. Normally distributed continuous variables were presented as mean (standard deviation, SD) and compared between groups using Student’s t-test. Skewed distributed continuous variables were presented as the median and interquartile range (P25, P75) and compared between groups using the Mann–Whitney U test. For categorical variables, frequencies (percentages) were presented, and differences between groups were tested using the chi-squared test or Fisher’s exact test. Overall survival time was calculated from the date of initial diagnosis to either death or the end of the study.

Univariate and multivariate Cox regression models were created to identify factors associated with mortality for anti-MDA5+ DM, presented as hazard ratios (HR) and 95% confidence intervals (CI). Survival analysis was performed using the Kaplan-Meier method, and differences in mortality rates between two groups of patients were compared using the Log-rank test. All analyses were 2-tailed and p values <0.05 were considered to indicate statistical significance.

## Results

**Figure 1:**
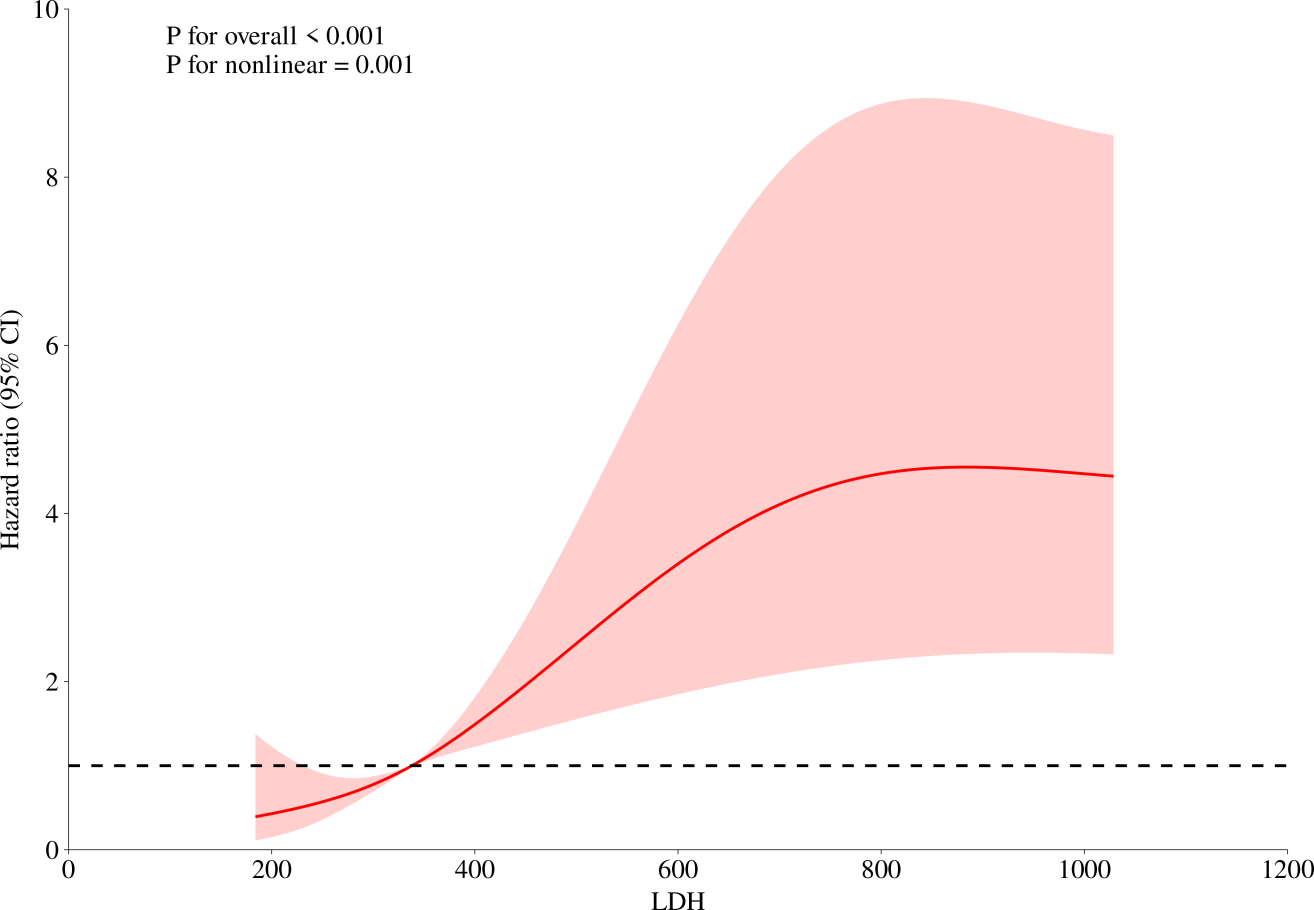
Nonlinear analysis of the relationship between LDH levels and mortality risk. This figure illustrates the relationship between LDH levels and mortality risk. The red curve in the figure represents the hazard ratio, which exhibits an inverted S-shaped trend as LDH levels increase, indicating a nonlinear relationship between LDH levels and overall mortality risk. The 95% confidence interval (CI) is also marked in the figure, shown as the shaded red area. The displayed P-values (P for overall < 0.001 and P for nonlinear = 0.001) suggest that this relationship is statistically significant.

This study collected data from 246 patients with anti-MDA5+ DM, aiming to explore the potential nonlinear relationship between serum LDH levels and the risk of death. To do this, we fitted a restricted cubic spline model with knots at the 10th, 50th, and 90th percentiles of the LDH distribution. A likelihood ratio test was used to compare the model with linear terms and the model with spline terms, in order to examine the nonlinear relationship between the two. The results showed a significant nonlinear relationship (P for nonlinear = 0.001), and the inflection point was visually identified from the plotted spline curve. Based on this inflection point, we divided the LDH levels into two groups: low LDH (≤ 338 U/L) and high LDH (>338 U/L) (**Figure 1**). Kaplan-Meier analysis and log-rank tests were used to assess the differences in overall survival between the two groups. Furthermore, we calculated the HR at different LDH levels and plotted the 95% CI to further quantify the relationship between LDH levels and the risk of death.

We collected data from 246 patients with anti-MDA5+ DM and divided them into a survival group (n=186) and a non-survival group (n=60) based on survival status. The clinical characteristics, laboratory results, and long-term prognosis of the two groups were compared. The results showed that there were no significant statistical differences between the survival and non-survival groups in terms of proximal muscle involvement, Rash, Gottron papules, heliotrope rash, V sign, cutaneous ulcers, shawl sign, periungual erythema, mechanic’s hands, ALT, ESR, positive anti-ARS antibodies, and positive antinuclear antibodies. The proportion of female patients in the survival group was higher (75.3% vs. 60%, P = 0.023), as was the disease course [3.0 (1.0, 6.0) vs. 1.0 (1.0, 3.0), P<0.001], and follow-up time [12.0 (8.0, 12.0) vs. 3.0 (2.0, 4.0), P<0.001], while the average age was lower in the survival group [52.0 (45.0, 59.8) vs. 59.5 (49.0, 66.0), P<0.001]. The incidence of arthritis was higher in the survival group (40.9% vs. 23.3%, P = 0.014). The ALT levels [63.5 (47.0, 89.2) vs. 49.6 (30.4, 80.0), P = 0.003], LDH levels [487.5 (330.0, 727.0) vs. 310.0 (245.5, 399.3), P<0.001], CK levels [109.5 (37.8, 263.0) vs. 60.0 (36.3, 129.8), P = 0.027], CRP levels [11.5 (4.9, 23.0) vs. 4.9 (2.9, 11.7), P<0.001], serum ferritin levels [1307.6 (962.0, 2000.0) vs. 1146.4 (422.6, 1307.6), P<0.001], and the incidence of RPILD (83.3% vs. 20.4%, P<0.001) were significantly higher in the non-survival group. These factors warrant further research and exploration regarding their impact on prognosis (**Table 1**).

**Table 1:** Clinical characteristics of patients grouped by survival status (Survival or Un-survival).

Variable	Total (*n* = 246)	Survival (n=186)	Un-survival (n=60)	*P*-value
Gender				**0.023**
Male, n (%)	70 (28.5)	46 (24.7)	24 (40.0)	
Female, n (%)	176 (71.5)	140 (75.3)	36 (60.0)	
Age, median (range), (years)	53.0 (47.0, 63.0)	52.0 (45.0, 59.8)	59.5 (49.0, 66.0)	<0.001*
Course of the disease, median (range), (months)	2.0 (1.0, 5.0)	3.0 (1.0, 6.0)	1.0 (1.0, 3.0)	<0.001*
Follow-up periods, median (range), (months)	12.0 (3.0, 12.0)	12.0 (8.0, 12.0)	3.0 (2.0, 4.0)	<0.001*
Proximal muscle involvement^#^, n (%)	112 (45.5)	88 (47.3)	24 (40.0)	0.323
Rash, n (%)	229 (93.1)	176 (94.6)	53 (88.3)	0.168
Gottron papule, n (%)	168 (68.3)	125 (67.2)	43 (71.7)	0.518
Heliotrope rash, n (%)	140 (56.9)	110 (59.1)	30 (50.0)	0.214
V sign, n (%)	89 (36.2)	70 (37.6)	19 (31.7)	0.403
Cutaneous ulcers, n (%)	34 (13.8)	24 (12.9)	10 (16.7)	0.463
Shawl sign, n (%)	55 (22.4)	39 (21.0)	16 (26.7)	0.357
Periungual erythema, n (%)	52 (21.1)	39 (21.0)	13 (21.7)	0.908
Arthritis, n (%)	90 (36.6)	76 (40.9)	14 (23.3)	**0.014**
Mechanic’s hands, n (%)	67 (27.2)	50 (26.9)	17 (28.3)	0.826
ALT, median (range), (U/L)	47.3 (29.0, 80.5)	46.0 (26.2, 81.4)	53.0 (36.0, 79.3)	0.154
AST, median (range), (U/L)	52.0 (32.9, 83.0)	49.6 (30.4, 80.0)	63.5 (47.0, 89.2)	**0.003**
LDH, median (range), (U/L)	421.1 ± 311.8	310.0 (245.5, 399.3)	487.5 (330.0, 727.0)	<0.001*
CK, median (range), (U/L)	63.0 (36.8, 158.0)	60.0 (36.3, 129.8)	109.5 (37.8, 263.0)	**0.027**
ESR, median (range), (mm/h)	42.0 ± 23.8	37.1 (23.0, 54.8)	42.0 (27.8, 62.0)	0.086
CRP, median (range), (mg/L)	5.9 (3.1, 12.2)	4.9 (2.9, 11.7)	11.5 (4.9, 23.0)	<0.001*
Serum ferritin (ng/mL)	1307.6 ± 2363.3	1146.4 (422.6, 1307.6)	1307.6 (962.0, 2000.0)	<0.001*
ANA positive, n (%)	129 (52.4)	98 (52.7)	31 (51.7)	0.890
Anti-Ro52 positive, n (%)	158 (64.2)	112 (60.2)	46 (76.7)	0.021*
Anti-ARS positive, n (%)	15 (6.1)	13 (7.0)	2 (3.3)	0.472
Anti-MDA5 antibody titer, n (%)				**0.002**
+	72 (29.3)	64 (34.4)	8 (13.3)	–
++	46 (18.7)	36 (19.4)	10 (16.7)	–
+++	128 (52.0)	86 (46.2)	42 (70.0)	–
ILD, n (%)	246 (100.0)	186 (100.0)	60 (100.0)	–
RPILD, n (%)	88 (35.8)	38 (20.4)	50 (83.3)	<0.001*

*The bolded numbers in the table represent statistical results with a p-value <0.05, indicating that the differences between groups are statistically significant. anti-MDA5+ DM, anti-melanoma differentiation-associated gene 5 positive dermatomyositis; RPILD, rapidly progressive interstitial lung disease; ALT, alanine aminotransferase; AST, aspartate aminotransferase; CK, creatine kinase; LDH, lactate dehydrogenase; ESR, erythrocyte sedimentation rate; CRP, C-reactive protein; ANA, antinuclear antibody; Anti-ARS, anti-aminoacyl-tRNA synthetase. ^#^Proximal muscle involvement denotes Proximal muscle weakness.

We collected data from 246 patients with anti-MDA5+ DM and divided them into two groups based on LDH levels: LDH ≤ 338 U/L group and LDH > 338 U/L group. The clinical characteristics, laboratory results, and long-term prognosis of the two groups were compared. The results showed no significant statistical differences between the two groups in terms of gender, age, proximal muscle involvement, rash, Gottron papules, heliotrope rash, V sign, cutaneous ulcers, shawl sign, periungual erythema, arthritis, Mechanic’s hands, ESR, positive anti-ARS antibodies, and positive antinuclear antibodies. The disease course in the LDH ≤ 338 U/L group was higher [3.0 (1.0, 6.0) vs 2.0 (1.0, 4.0), P = 0.026] compared to the LDH > 338 U/L group. The ALT levels [64.0 (32.0, 121.0) vs 40.0 (23.0, 66.5), P<0.001], AST levels [75.0 (47.5, 134.0) vs 40.0 (26.0, 60.6), P<0.001], CK levels [107.0 (42.0, 208.8) vs 50.0 (35.5, 100.5), P<0.001], CRP levels [8.5 (3.5, 17.6) vs 4.7 (2.7, 10.2), P<0.001], and serum ferritin levels [1307.6 (679.8, 1565.5) vs 1001.0 (391.2, 1307.6), P<0.001] were significantly higher in the LDH > 338 U/L group compared to the LDH ≤ 338 U/L group. Furthermore, the positivity rate of Anti-Ro52 antibodies (70.7% vs 57.7%, P = 0.033), the incidence of RPILD (42.3% vs 29.3%, P = 0.033), and the mortality rate (35.0% vs 13.8%, P<0.001) were significantly higher in the LDH > 338 U/L group compared to the LDH ≤ 338 U/L group. These factors warrant further research and exploration regarding their impact on prognosis (**Table 2**).

**Table 2:** Clinical characteristics grouped by LDH Levels.

Variable	LDH ≦ 338U/L (n=123)	LDH > 338U/L (n=123)	*P*-value
Gender			0.572
Male, n (%)	33 (26.8)	37 (30.1)	
Female, n (%)	90 (73.2)	86 (69.9)	
Age, median (range), (years)	54.0 (47.0, 63.0)	52.0 (47.0, 62.0)	0.453
Course of the disease, median (range), (months)	3.0 (1.0, 6.0)	2.0 (1.0, 4.0)	**0.026**
Proximal muscle involvement^#^, n (%)	52 (42.3)	60 (48.8)	0.306
Rash, n (%)	118 (95.9)	111 (90.2)	0.078
Gottron papule, n (%)	91 (74.0)	77 (62.6)	0.055
Heliotrope rash, n (%)	73 (59.4)	67 (54.5)	0.440
V sign, n (%)	47 (38.2)	42 (34.2)	0.507
Cutaneous ulcers, n (%)	15 (12.2)	19 (15.5)	0.460
Shawl sign, n (%)	30 (24.4)	25 (20.3)	0.444
Periungual erythema, n (%)	24 (19.5)	28 (22.7)	0.532
Arthritis, n (%)	47 (38.2)	43 (35.0)	0.596
Mechanic’s hands, n (%)	39 (31.7)	28 (22.8)	0.115
ALT, median (range), (U/L)	40.0 (23.0, 66.5)	64.0 (32.0, 121.0)	<0.001*
AST, median (range), (U/L)	40.0 (26.0, 60.6)	75.0 (47.5, 134.0)	<0.001*
CK, median (range), (U/L)	50.0 (35.5, 100.5)	107.0 (42.0, 208.8)	<0.001*
ESR, median (range), (mm/h)	37.0 (21.5, 56.0)	42.0 (26.9, 54.5)	0.236
CRP, median (range), (mg/L)	4.7 (2.7, 10.2)	8.5 (3.5, 17.6)	<0.001*
Serum ferritin (ng/mL)	1001.0 (391.2, 1307.6)	1307.6 (679.8, 1565.5)	<0.001*
ANA positive, n (%)	64 (52.0)	65 (52.9)	0.898
Anti-Ro52 positive, n (%)	71 (57.7)	87 (70.7)	0.033*
Anti-ARS positive, n (%)	9 (7.3)	6 (4.9)	0.424
Anti-MDA5 antibody titer, n (%)			0.021*
+	45 (36.6)	27 (22.0)	–
++	24 (19.5)	22 (17.9)	–
+++	54 (43.9)	74 (60.2)	–
ILD, n (%)	123 (100.0)	123 (100.0)	–
RPILD, n (%)	36 (29.3)	52 (42.3)	0.033*
Death, n (%)	17 (13.8)	43 (35.0)	<0.001*

*The bolded numbers in the table represent statistical results with a p-value <0.05, indicating that the differences between groups are statistically significant. anti-MDA5+ DM, anti-melanoma differentiation-associated gene 5 positive dermatomyositis; RPILD, rapidly progressive interstitial lung disease; ALT, alanine aminotransferase; AST, aspartate aminotransferase; CK, creatine kinase; LDH, lactate dehydrogenase; ESR, erythrocyte sedimentation rate; CRP, C-reactive protein; ANA, antinuclear antibody; Anti-ARS, anti-aminoacyl-tRNA synthetase. ^#^Proximal muscle involvement denotes Proximal muscle weakness.

In the 246 anti-MDA5+ DM patients, univariate and multivariate Cox regression analyses were performed to assess the risk factors for mortality. The results indicated that age, course of the disease, LDH>338 U/L, CRP levels, male, anti-Ro52 antibody positivity, increased anti-MDA5 antibody titers, and the presence of RPILD were significantly associated with poor overall prognosis in the patients. Subsequently, all meaningful variables used as covariates were included in the multivariate analysis. Multivariate regression analysis showed that elevated LDH levels (>338 U/L) and the presence of RPILD were associated with poor prognosis, with hazard ratios of 2.253 (95% CI 1.258, 4.035, P = 0.006) and 10.293 (95% CI 4.683, 22.623, P < 0.001), respectively (**Table 3**).

**Table 3:** Prognostic factors for survival in 246 anti-MDA5+ DM patients.

Variable	Univariate analysis		Multivariate analysis	
	HR(95% CI)	*P*-value	HR(95% CI)	*P*-value
Age	1.043 (1.020 ~ 1.067)	**<0.001**	1.024 (0.998 ~ 1.051)	0.068
Course of the disease	0.918 (0.848 ~ 0.993)	**0.033**	0.992 (0.946 ~ 1.040)	0.741
LDH				
LDH ≦ 338U/L	1.000 (Reference)		1.000 (Reference)	
LDH > 338U/L	2.793 (1.593~ 4.899)	**<0.001**	2.253 (1.258~ 4.035)	**0.006**
CRP	1.020 (1.013 ~ 1.028)	**<0.001**	1.008 (0.999 ~ 1.017)	0.091
Sex				
Female	1.000 (Reference)		1.000 (Reference)	
Male	1.798 (1.072 ~ 3.015)	**0.026**	1.207 (0.697 ~ 2.088)	0.502
Arthritis				
No	1.000 (Reference)		1.000 (Reference)	
Yes	0.501 (0.275 ~ 0.911)	**0.023**	0.494 (0.268 ~ 0.912)	**0.024**
Anti-Ro52 antibody				
–	1.000 (Reference)		1.000 (Reference)	
+	2.085 (1.146 ~ 3.795)	**0.016**	0.793 (0.406 ~ 1.549)	0.497
Anti-MDA5 antibody titer				
+	1.000 (Reference)		1.000 (Reference)	
++	2.015 (0.795 ~ 5.107)	0.140	1.113 (0.432 ~ 2.863)	0.825
+++	3.273 (1.536 ~ 6.975)	**0.002**	1.636 (0.742 ~ 3.607)	0.223
RPILD				
No	1.000 (Reference)		1.000 (Reference)	
Yes	13.357 (6.730 ~ 26.510)	**<0.001**	10.293 (4.683 ~ 22.623)	**<0.001**

The bolded numbers in the table represent statistical results with a p-value <0.05. LDH, lactate dehydrogenase; CRP, C-reactive protein; anti-MDA5, anti-melanoma differentiation-associated gene 5; RPILD, rapidly progressive interstitial lung disease.

**Figure 2:**
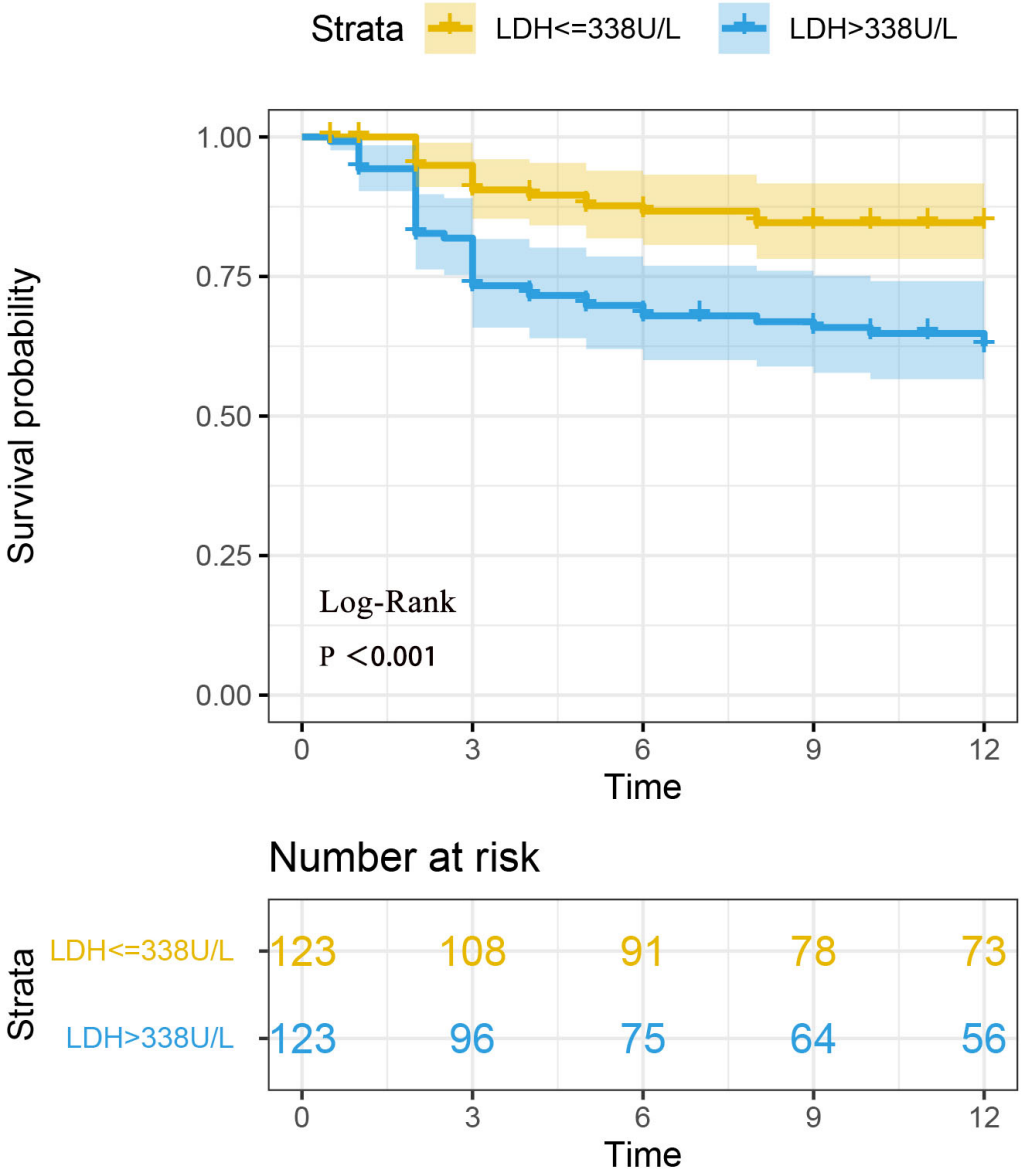
Survival analysis of two groups of anti-MDA5+ DM patients based on LDH levels. Survival analysis revealed a statistically significant difference in mortality between the two groups of patients with different LDH levels (Log-rank P < 0.001).

The follow-up time for 246 patients ranged from 0.5 months to 12 months, with a median follow-up time of 12 months. There were a total of 60 patients died during the follow-up period (one year). The survival times of the two groups of patients were compared. The survival analysis showed a significant difference in mortality between the two groups with different LDH levels (Log-Rank P < 0.001). The Kaplan-Meier curve is shown in **Figure 2**.

## Discussion

This study found that serum LDH levels have important value in the clinical features and prognosis assessment of anti-MDA5+ DM patients. Through the analysis of 246 patients, the study showed that patients with LDH > 338 U/L had significantly higher levels of liver enzymes (ALT, AST), CK, CRP, and serum ferritin. Additionally, the positive rate of anti-Ro52 antibodies, the incidence of RPILD, and the one-year mortality rate (35.0% vs 13.8%) were also significantly higher compared to the LDH ≤ 338 U/L group. Multivariate regression analysis further emphasized the independent predictive role of elevated LDH levels (> 338 U/L) and RPILD, which increased the risk of death by 2.253 times and 10.293 times, respectively. Notably, LDH showed a non-linear, inverted S-shaped relationship with overall mortality risk (non-linear P = 0.001), suggesting that the clinical significance of LDH may not be limited to a single threshold, and its dynamic changes or monitoring may provide more valuable references for disease progression.

Currently, the treatment of anti-MDA5+ DM patients has yet to achieve optimal outcomes, and prognostic biomarkers are crucial for patient stratification and appropriate management (24). A recent study proposed the “FLAIR score,” which consists of five key indicators: ferritin levels, LDH levels, anti-MDA5 antibody grading, HRCT imaging score, and RPILD/non-RPILD, to predict mortality in patients with amyopathic dermatomyositis (ADM)-associated ILD (25). Additionally, KL-6, interleukin (IL)-15, serum chitinase-3-like-1 protein (YKL-40), IL-6, interferon-α, and CD4+CXCR4+ T cells have also been reported as prognostic biomarkers in anti-MDA5+ DM patients, although none have been widely accepted so far (26–29). Our study elucidates the close association between elevated serum LDH levels and poor prognosis in anti-MDA5+ DM patients, revealing significant prognostic differences based on LDH levels and the presence of RPILD. The findings suggest that patients with LDH > 338 U/L or those with RPILD have a significantly higher risk of mortality, and dynamic monitoring of LDH levels can help identify the prognostic characteristics of these high-risk patients early. From the perspective of clinical practice, these findings provide important evidence for the individualized management of anti-MDA5+ DM patients. LDH, as an easily accessible inflammatory marker, combined with the presence of RPILD, can effectively screen for patients who require intensified interventions. For example, in patients with LDH > 338 U/L or those with RPILD, clinicians can prioritize initiating enhanced immunosuppressive treatments (such as a combination of corticosteroids and immunoglobulin) or multidisciplinary collaboration (such as early respiratory support and lung protection strategies) to delay lung disease progression and improve survival outcomes. Furthermore, the widespread applicability of LDH makes it an economically efficient risk stratification tool in resource-limited areas, particularly suitable for medical settings where complex biomarkers cannot be frequently tested, providing a practical and feasible prognostic assessment solution for primary care hospitals. By incorporating dynamic monitoring of LDH into routine clinical pathways, early warning and precise interventions for high-risk patients can be achieved, thus optimizing overall treatment strategies (3, 30).

Previous studies have consistently reported that the mortality rate of anti-MDA5+ DM-associated ILD patients exceeds 60%, particularly in East Asian populations, such as in China (31). Most deaths occur within the first 3 months (2). It is widely believed that excessive inflammatory responses are associated with an increased risk of RPILD and all-cause mortality (32). Excessive inflammation plays a crucial role in the onset and progression of anti-MDA5+ DM-associated ILD. Uncontrolled excessive inflammation and persistent immune activation lead to severe lung damage and RPILD, which may resemble the “cytokine storm” phenomenon observed in COVID-19 (33). A study aimed to identify the best prediction model for the 3-month mortality risk in patients with anti-MDA5+DM-associated ILD, revealing that RPILD, ESR, serum albumin level, age, CRP, AST, NLR, and LDH are eight key variables with significant predictive importance. Most of these variables are inflammatory markers reflecting a high inflammatory state, with LDH being one of the important risk factors (30). Previous studies have shown that poor prognosis in DM patients is associated with certain factors, including advanced age and elevated levels of serum ferritin, LDH, NLR, and CRP, which is consistent with the findings of our study (31). LDH is easily accessible and observable in clinical settings for DM patients, making it of significant practical relevance. Another large-scale single-center cohort study in China proposed a model called the “FLAIR score,” which combines ferritin(<636 ng/mL, 0; ≥636 ng/mL, 2), LDH(<355 U/L, 0; ≥355 U/L, 2), anti-MDA5 antibody(negative, 0; +, 2; ++, 3; +++, 4), HRCT imaging scores(<133, 0; ≥133, 3), and RPILD(non-RPILD, 0; RPILD, 2) to predict the mortality of amyopathic dermatomyositis (ADM)-associated ILD (25). Furthermore, elevated LDH not only suggests poor prognosis but also has clinical significance in other aspects. One study evaluated the predictive factors for relapse in polymyositis/dermatomyositis (PM/DM), revealing that serum LDH levels exceeding 450U/L could predict relapse in PM/DM-related ILD patients, with an area under the curve of 0.718 (34). In conclusion, LDH is considered a prognostic indicator for poor outcomes in patients with anti-MDA5+ DM.

However, different studies have used varying prognostic cutoff values for LDH. A study on Chinese anti-MDA5+ DM patients with RPILD and its prognostic factors reported that elevated LDH levels were associated with RPILD and mortality, with a threshold of 356.15 U/L (3). Another study in anti-MDA5+ DM patients showed that LDH levels were positively correlated with RPILD and mortality risk, but the threshold was not defined (5). A recent cohort study indicated that survivors of MDA5+ DM-ILD had significantly lower serum LDH levels than those who died, and LDH (>355 U/L) may be an independent high-risk factor for poor prognosis (25). Our threshold of 338 U/L is consistent with these studies and further optimized risk stratification through nonlinear analysis (inverse S-shaped relationship, nonlinear P = 0.001). Using this threshold, we were able to classify patients into low-risk (LDH ≤ 338 U/L) and high-risk (LDH>338 U/L) groups, with the high-risk group having a significantly higher mortality rate (35.0% vs. 13.8%, P<0.001) and LDH >338 U/L is an independent predictor of mortality (hazard ratio 2.253), similar to the FLAIR model by Lian et al. (25). This suggests that in clinical practice, LDH>338 U/L can serve as a simple and rapid bedside indicator to help physicians identify patients who may require closer monitoring and more active intervention. However, we acknowledge that thresholds may vary depending on the population, testing methods, and laboratory standards, and therefore our results require external validation. We suggest that future multicenter studies use standardized protocols to validate this threshold and explore the development of a composite scoring system incorporating other biomarkers (such as serum ferritin) to improve predictive accuracy.

LDH is an intracellular enzyme released into the bloodstream during cell damage or death, so its elevation usually reflects tissue destruction and an inflammatory state. In anti-MDA5+ DM, elevated LDH may be associated with the disease pathophysiology through the following mechanisms. First, the core features of dermatomyositis include muscle inflammation and skin lesions (35). LDH is a stable cytoplasmic enzyme present in all cells. When cells are damaged or undergo necrosis, the permeability of the cell membrane increases, leading to the release of LDH (36). Anti-MDA5 antibodies may induce muscle cell apoptosis and necrosis through the activation of the type I interferon pathway, resulting in LDH release. Second, anti-MDA5+ DM is often associated with RPILD, characterized by rapid damage and fibrosis of alveolar epithelial cells and vascular endothelial cells. Recent studies have confirmed that activated macrophages play a role in the occurrence and progression of pulmonary fibrosis in various ways, such as by inducing neutrophil activation and triggering the formation of neutrophil extracellular traps (37, 38). The specific mechanism by which LDH participates in pulmonary fibrosis is not fully understood, but studies suggest that LDH is a marker enzyme of macrophages, and its activity can serve as an indicator of macrophage activation (36). High LDH concentrations are consistently associated with severe pulmonary fibrosis and lung injury (39). Our data show that the LDH>338 U/L group has a higher incidence of RPILD (42.3% vs. 29.3%, P = 0.033), and in multivariable analysis, RPILD is a strong predictor of mortality (hazard ratio 10.293), which supports the role of LDH as an alternative biomarker for lung injury. Finally, anti-MDA5 antibodies may activate the innate immune response (such as MDA5 recognizing viral RNA mimics), leading to macrophage and lymphocyte activation and the release of pro-inflammatory cytokines (e.g., IL-6, TNF-α) (35). These factors can induce widespread cell death, further elevating LDH levels. Additionally, high serum ferritin levels (higher in the LDH>338 U/L group in this study) suggest that macrophage activation may exacerbate tissue damage. These mechanisms are hypothesized based on the current understanding of the pathophysiology of anti-MDA5+ DM, but further experimental research is needed for validation.

Although we performed a multivariable Cox regression analysis, adjusting for variables such as age, gender, key laboratory markers, potential confounding factors such as treatment regimens (e.g., immunosuppressive therapy, corticosteroid dosage) and comorbidities were not fully controlled. In anti-MDA5+ DM patients, glucocorticoids may indirectly lower LDH levels by inhibiting inflammatory responses and cellular damage. Conversely, if patients do not respond well to treatment, LDH levels may remain elevated, indicating poor prognosis. Moreover, the type and dosage of immunosuppressants may vary due to patient heterogeneity. In our study, we did not systematically collect data on treatment doses and specific regimens. We acknowledge that differences in treatment regimens may still act as residual confounding factors, potentially leading to an underestimation or overestimation of the association between LDH and prognosis. Future prospective studies should systematically document treatment data and further control for these confounders through sensitivity analyses. The impact of comorbidities should not be overlooked. LDH is widely distributed in various tissues (such as the liver, heart, muscles, and lungs), and therefore, comorbidities like liver diseases (e.g., fatty liver or hepatitis), heart failure, or chronic kidney disease may independently lead to elevated LDH levels. For instance, liver comorbidities might indirectly affect LDH levels by elevating ALT and AST levels, and our data show significantly higher ALT and AST levels in the LDH>338 U/L group, suggesting that liver injury may partially mediate the association between LDH and prognosis. In the multivariable analysis, we partially controlled for this confounding factor by adjusting for liver function markers (ALT, AST), but could not completely eliminate its influence. In conclusion, these factors may complicate the relationship between LDH and mortality.

In this study, some patients were simultaneously positive for both anti-MDA5 and anti-ARS antibodies. In clinical practice, when patients test positive for multiple myositis-specific antibodies, they are typically categorized based on their most representative clinical manifestations and antibody types. Anti-MDA5 antibodies are strongly associated with RPILD and have a poor prognosis, which is the central focus of our study. If patients who are also positive for anti-ARS antibodies were separately classified as having “anti-synthetase syndrome,” it would dilute the clinical characteristics of the anti-MDA5+ DM cohort, particularly in analyses related to RPILD and mortality risk. This study classifies patients who test positive for anti-ARS antibodies as anti-MDA5+ DM patients, based on the research objectives, the predominance of clinical phenotypes, and the consistency of statistical analysis. This approach helps to more clearly reveal the independent role of anti-MDA5 antibodies in RPILD and mortality risk, without interference from other antibody phenotypes.

The strengths of this study are attributed to its large multicenter sample size (246 consecutive cases) and the combination of systematic analytical methods. By comprehensively evaluating clinical features, laboratory markers, and long-term prognosis, the study not only revealed the association between LDH and the prognosis of anti-MDA5+ DM but also controlled for potential confounding factors through multivariate regression models, enhancing the reliability of the results. Furthermore, as LDH is a routine diagnostic test, its clinical applicability further increases the practical value of the study. However, the study also has some limitations. First, the retrospective design may introduce selection bias, and future multicenter prospective studies are needed to validate the generalizability of the conclusions. Second, although some confounding factors have been adjusted for, differences in treatment regimens, comorbidities, and other variables were not fully included in the analysis, which may affect the accuracy of the results. Third, the LDH threshold (338 U/L) was defined based on the current cohort, and its applicability in different populations requires external validation. Fourth, the follow-up period was relatively short, and there was a lack of assessment of long-term survival conditions. Finally, the biological mechanisms underlying elevated LDH in anti-MDA5+ DM (such as the contributions of tissue damage and systemic inflammation) have not been clarified and require further investigation through basic research.

## Data Availability

The original contributions presented in the study are included in the article. Further inquiries can be directed to the corresponding author.

## References

[1] Xu L You H Wang L Lv C Yuan F Li J . Identification of three different phenotypes in anti-melanoma differentiation-associated gene 5 antibody-positive dermatomyositis patients: implications for prediction of rapidly progressive interstitial lung disease. Arthritis Rheumatol (Hoboken N.J.). (2023) 75:609–19. doi: 10.1002/art.42308, PMID: 35849805

[2] Allenbach Y Uzunhan Y Toquet S Leroux G Gallay L Marquet A . Different phenotypes in dermatomyositis associated with anti-MDA5 antibody: Study of 121 cases. Neurology. (2020) 95:e70–e8. doi: 10.1212/WNL.0000000000009727, PMID: 32487712 PMC7371381

[3] Li M Zhao X Liu B Zhao Y Li X Ma Z . Predictors of rapidly progressive interstitial lung disease and prognosis in Chinese patients with anti-melanoma differentiation-associated gene 5-positive dermatomyositis. Front Immunol. (2023) 14:1209282. doi: 10.3389/fimmu.2023.1209282, PMID: 37691917 PMC10483132

[4] Jin Q Fu L Yang H Chen X Lin S Huang Z . Peripheral lymphocyte count defines the clinical phenotypes and prognosis in patients with anti-MDA5-positive dermatomyositis. J Internal Med. (2023) 293:494–507. doi: 10.1111/joim.13607, PMID: 36682032

[5] Zuo Y Ye L Chen F Shen Y Lu X Wang G . Different multivariable risk factors for rapid progressive interstitial lung disease in anti-MDA5 positive dermatomyositis and anti-synthetase syndrome. Front Immunol. (2022) 13:845988. doi: 10.3389/fimmu.2022.845988, PMID: 35320936 PMC8936070

[6] Shi Y You H Liu C Qiu Y Lv C Zhu Y . Elevated serum B-cell activator factor levels predict rapid progressive interstitial lung disease in anti-melanoma differentiation associated protein 5 antibody positive dermatomyositis. Orphanet J rare Dis. (2024) 19:170. doi: 10.1186/s13023-024-03153-6, PMID: 38637830 PMC11027411

[7] Liu T Li W Zhang Z Jiang T Fei Y Huang J . Neutrophil-to-lymphocyte ratio is a predictive marker for anti-MDA5 positive dermatomyositis. BMC pulmonary Med. (2022) 22:316. doi: 10.1186/s12890-022-02106-8, PMID: 35978395 PMC9382756

[8] Gono T Kawaguchi Y Satoh T Kuwana M Katsumata Y Takagi K . Clinical manifestation and prognostic factor in anti-melanoma differentiation-associated gene 5 antibody-associated interstitial lung disease as a complication of dermatomyositis. Rheumatol (Oxford England). (2010) 49:1713–9. doi: 10.1093/rheumatology/keq149, PMID: 20498012

[9] Fujiki Y Kotani T Isoda K Ishida T Shoda T Yoshida S . Evaluation of clinical prognostic factors for interstitial pneumonia in anti-MDA5 antibody-positive dermatomyositis patients. Modern Rheumatol. (2018) 28:133–40. doi: 10.1080/14397595.2017.1318468, PMID: 28490218

[10] Gono T Sato S Kawaguchi Y Kuwana M Hanaoka M Katsumata Y . Anti-MDA5 antibody, ferritin and IL-18 are useful for the evaluation of response to treatment in interstitial lung disease with anti-MDA5 antibody-positive dermatomyositis. Rheumatol (Oxford England). (2012) 51:1563–70. doi: 10.1093/rheumatology/kes102, PMID: 22589330

[11] So J So H Wong VT Ho R Wu TY Wong PC . Predictors of rapidly progressive interstitial lung disease and mortality in patients with autoantibodies against melanoma differentiation-associated protein 5 dermatomyositis. Rheumatol (Oxford England). (2022) 61:4437–44. doi: 10.1093/rheumatology/keac094, PMID: 35157042

[12] Ye Y Fu Q Wang R Guo Q Bao C . Serum KL-6 level is a prognostic marker in patients with anti-MDA5 antibody-positive dermatomyositis associated with interstitial lung disease. J Clin Lab Anal. (2019) 33:e22978. doi: 10.1002/jcla.22978, PMID: 31301087 PMC6805307

[13] Liang J Wan L Yao Y Cui X He Y Li S . An externally validated clinical-laboratory nomogram for myocardial involvement in adult idiopathic-inflammatory-myopathy patients. Clin Rheumatol. (2024) 43:1959–69. doi: 10.1007/s10067-024-06948-x, PMID: 38587715 PMC11111495

[14] Liu AC Yang Y Li MT Jia Y Chen S Ye S . Macrophage activation syndrome in systemic lupus erythematosus: a multicenter, case-control study in China. Clin Rheumatol. (2018) 37:93–100. doi: 10.1007/s10067-017-3625-6, PMID: 28409239

[15] Li J Jiang JJ Wang CY Jian S Zhou Y Ma MS . Clinical features and prognosis of patients with thrombotic thrombocytopenic purpura associated with systemic lupus erythematosus: a review of 25 cases. Ital J Pediatr. (2019) 45:55. doi: 10.1186/s13052-019-0641-y, PMID: 31036039 PMC6489191

[16] Cheng L Xu L Xu Y Yuan F Li J Wu M . Gender differences in patients with anti-MDA5-positive dermatomyositis: a cohort study of 251 cases. Clin Rheumatol. (2024) 43:339–47. doi: 10.1007/s10067-023-06816-0, PMID: 37985533

[17] Wang L Lv C You H Xu L Yuan F Li J . Rapidly progressive interstitial lung disease risk prediction in anti-MDA5 positive dermatomyositis: the CROSS model. Front Immunol. (2024) 15:1286973. doi: 10.3389/fimmu.2024.1286973, PMID: 38361940 PMC10867574

[18] Wang G Yan D Weng C Xue L Liu Z . Clinical features and prognosis of anti-MDA5-positive dermatomyositis with coexistent anti-aminoacyl-tRNA synthetase antibodies. Clin Rheumatol. (2025) 44:767–74. doi: 10.1007/s10067-024-07298-4, PMID: 39751977

[19] Bohan A Peter JB . Polymyositis and dermatomyositis (second of two parts). N Engl J Med. (1975) 292:403–7. doi: 10.1056/NEJM197502202920807, PMID: 1089199

[20] Bohan A Peter JB . Polymyositis and dermatomyositis (first of two parts). N Engl J Med. (1975) 292:344–7. doi: 10.1056/NEJM197502132920706, PMID: 1090839

[21] Schaefer-Prokop C Prokop M Fleischmann D Herold C . High-resolution CT of diffuse interstitial lung disease: key findings in common disorders. Eur Radiol. (2001) 11:373–92. doi: 10.1007/s003300000648, PMID: 11288840

[22] Abe Y Kusaoi M Tada K Yamaji K Tamura N . Successful treatment of anti-MDA5 antibody-positive refractory interstitial lung disease with plasma exchange therapy. Rheumatol (Oxford England). (2020) 59:767–71. doi: 10.1093/rheumatology/kez357, PMID: 31504956

[23] Won Huh J Soon Kim D Keun Lee C Yoo B Bum Seo J Kitaichi M . Two distinct clinical types of interstitial lung disease associated with polymyositis-dermatomyositis. Respir Med. (2007) 101:1761–9. doi: 10.1016/j.rmed.2007.02.017, PMID: 17428649

[24] Duremala F Tiniakou E Andrews J . Epidemiology of myositis. Curr Opin Rheumatol. (2025) 37:121–7. doi: 10.1097/BOR.0000000000001076, PMID: 39655458 PMC13050537

[25] Lian X Zou J Guo Q Chen S Lu L Wang R . Mortality risk prediction in amyopathic dermatomyositis associated with interstitial lung disease: the FLAIR model. Chest. (2020) 158:1535–45. doi: 10.1016/j.chest.2020.04.057, PMID: 32428508

[26] Jiang L Wang Y Peng Q Shu X Wang G Wu X . Serum YKL-40 level is associated with severity of interstitial lung disease and poor prognosis in dermatomyositis with anti-MDA5 antibody. Clin Rheumatol. (2019) 38:1655–63. doi: 10.1007/s10067-019-04457-w, PMID: 30739212

[27] Nara M Komatsuda A Omokawa A Togashi M Okuyama S Sawada K . Serum interleukin 6 levels as a useful prognostic predictor of clinically amyopathic dermatomyositis with rapidly progressive interstitial lung disease. Modern Rheumatol. (2014) 24:633–6. doi: 10.3109/14397595.2013.844390, PMID: 24252021

[28] Horai Y Koga T Fujikawa K Takatani A Nishino A Nakashima Y . Serum interferon-α is a useful biomarker in patients with anti-melanoma differentiation-associated gene 5 (MDA5) antibody-positive dermatomyositis. Modern Rheumatol. (2015) 25:85–9. doi: 10.3109/14397595.2014.900843, PMID: 24716595

[29] Wu W Guo L Fu Y Wang K Zhang D Xu W . Interstitial lung disease in anti-MDA5 positive dermatomyositis. Clin Rev Allergy Immunol. (2021) 60:293–304. doi: 10.1007/s12016-020-08822-5, PMID: 33405101

[30] Li H Zou R Xin H He P Xi B Tian Y . Mortality risk prediction in patients with antimelanoma differentiation-associated, gene 5 antibody-positive, dermatomyositis-associated interstitial lung disease: algorithm development and validation. J Med Internet Res. (2025) 27:e62836. doi: 10.2196/62836, PMID: 39908093 PMC11840371

[31] Yang B Liu S Qian Z Tong Z . Predicting the death of patients with anti-melanoma differentiation-associated protein-5-positive dermatomyositis-associated interstitial lung disease: A systematic review and meta-analysis. Modern Rheumatol. (2024) 34:541–50. doi: 10.1093/mr/road042, PMID: 37364274

[32] Lv C You H Xu L Wang L Yuan F Li J . Coexistence of anti-ro52 antibodies in anti-MDA5 antibody-positive dermatomyositis is highly associated with rapidly progressive interstitial lung disease and mortality risk. J Rheumatol. (2023) 50:219–26. doi: 10.3899/jrheum.220139, PMID: 35705235

[33] Yongzhi X . COVID-19-associated cytokine storm syndrome and diagnostic principles: an old and new Issue. Emerging Microbes infections. (2021) 10:266–76. doi: 10.1080/22221751.2021.1884503, PMID: 33522893 PMC7894425

[34] Kishaba T Yano H Itagane M Sudo K Nagano H Kinjo M. Predictors of relapse of polymyositis/dermatomyositis associated interstitial lung disease. J Thorac Dis. (2024) 16:4229–37. doi: 10.21037/jtd-23-1736, PMID: 39144313 PMC11320281

[35] Lu X Peng Q Wang G . Anti-MDA5 antibody-positive dermatomyositis: pathogenesis and clinical progress. Nat Rev Rheumatol. (2024) 20:48–62. doi: 10.1038/s41584-023-01054-9, PMID: 38057474

[36] Xu X Huang Q Mao Y Cui Z Li Y Huang Y . Immunomodulatory effects of Bacillus subtilis (natto) B4 spores on murine macrophages. Microbiol Immunol. (2012) 56:817–24. doi: 10.1111/j.1348-0421.2012.00508.x, PMID: 22957751

[37] Zhang S Jia X Zhang Q Zhang L Yang J Hu C . Neutrophil extracellular traps activate lung fibroblast to induce polymyositis-related interstitial lung diseases via TLR9-miR-7-Smad2 pathway. J Cell Mol Med. (2020) 24:1658–69. doi: 10.1111/jcmm.14858, PMID: 31821687 PMC6991674

[38] Seto N Torres-Ruiz JJ Carmona-Rivera C Pinal-Fernandez I Pak K Purmalek MM . Neutrophil dysregulation is pathogenic in idiopathic inflammatory myopathies. JCI Insight. (2020) 5:e134189. doi: 10.1172/jci.insight.134189, PMID: 31945019 PMC7098779

[39] van Krugten M Cobben NA Lamers RJ van Dieijen-Visser MP Wagenaar SS Wouters EF . Serum LDH: a marker of disease activity and its response to therapy in idiopathic pulmonary fibrosis. Neth J Med. (1996) 48:220–3. doi: 10.1016/0300-2977(95)00074-7, PMID: 8710042

